# Fibroblast-Like Synoviocytes Induce Calcium Mineral Formation and Deposition

**DOI:** 10.1155/2014/812678

**Published:** 2014-05-20

**Authors:** Yubo Sun, David R. Mauerhan, Atiya M. Franklin, Natalia Zinchenko, Harry James Norton, Edward N. Hanley, Helen E. Gruber

**Affiliations:** ^1^Department of Orthopedic Surgery, Carolinas Medical Center, P.O. Box 32861, Charlotte, NC 28232, USA; ^2^Dickson Advanced Analytics Group, Carolinas Medical Center, P.O. Box 32861, Charlotte, NC 28232, USA

## Abstract

Calcium crystals are present in the synovial fluid of 65%–100% patients with osteoarthritis (OA) and 20%–39% patients with rheumatoid arthritis (RA). This study sought to investigate the role of fibroblast-like synoviocytes (FLSs) in calcium mineral formation. We found that numerous genes classified in the biomineral formation process, including bone gamma-carboxyglutamate (gla) protein/osteocalcin, runt-related transcription factor 2, ankylosis progressive homolog, and parathyroid hormone-like hormone, were differentially expressed in the OA and RA FLSs. Calcium deposits were detected in FLSs cultured in regular medium in the presence of ATP and FLSs cultured in chondrogenesis medium in the absence of ATP. More calcium minerals were deposited in the cultures of OA FLSs than in the cultures of RA FLSs. Examination of the micromass stained with nonaqueous alcoholic eosin indicated the presence of birefringent crystals. Phosphocitrate inhibited the OA FLSs-mediated calcium mineral deposition. These findings together suggest that OA FLSs are not passive bystanders but are active players in the pathological calcification process occurring in OA and that potential calcification stimuli for OA FLSs-mediated calcium deposition include ATP and certain unidentified differentiation-inducing factor(s). The OA FLSs-mediated pathological calcification process is a valid target for the development of disease-modifying drug for OA therapy.

## 1. Introduction

Basic calcium phosphate (BCP) crystals and calcium pyrophosphate dihydrate (CPPD) crystals are the two most common forms of articular crystals. Earlier studies find that these calcium crystals are associated with end-stage osteoarthritis (OA) in approximately 60% of cases [1, 2]. Recent studies, however, find that these calcium crystals are associated with end-stage OA in all cases and that the extent of cartilage calcification correlates with the histological grade of cartilage degeneration and clinical symptoms [3–6]. In addition, studies found that more calcium minerals are formed and deposited in the cultures of OA chondrocytes and meniscal cells than in the cultures of normal chondrocytes and meniscal cells [4, 6]. These findings indicate that pathological calcification is an active component of the OA disease process.

Increasing experimental evidence indicates that these calcium crystals may play a role in the disease process of OA. Injection of calcium crystals into the knee joints of dogs and mice induced severe inflammatory response [7, 8]. Calcium crystals added into cell culture induced cell mitogenesis and the production of matrix metalloproteinases (MMPs), nitric oxide, and inflammatory cytokines including interleukin-1*β* and cyclooxygenase-2 [9–13]. Phosphocitrate (PC), a potent calcification inhibitor, inhibited meniscal calcification in Hartley guinea pigs and the decrease in meniscal calcification was accompanied by a significant reduction in the severity of cartilage degeneration [14]. It is believed that PC exerts its disease-modifying activity on OA, at least in part, by inhibiting the formation of articular calcium crystals [15].

Because the most striking pathological changes are found in articular cartilage, OA is originally considered as a cartilage disease. OA fibroblast-like synoviocytes (FLSs) have traditionally been considered to be normal and were used as controls in studies investigating the cellular alterations occurring in rheumatoid arthritis (RA) FLSs. However, it has been gradually realized that OA is a whole joint disease. A low degree of synovium inflammation is common in OA [16–18]. OA synovium and OA FLS display differential gene expressions compared to RA or normal synovium and FLSs [19–21]. These observations and findings indicate that OA synovium or OA FLSs may not be passive bystanders in the initiation or progression of OA.

The presence of calcium crystals in OA articular cartilage is well-recognized and the chondrocyte-mediated calcification process has been studied extensively. However, calcium crystals were not only detected in the cartilage and synovial fluid but also detected in the synovial membranes of OA patients [8, 22]. In addition, 100% of OA patients with synovial calcium crystals have synovial tissue fragments, whereas only 63% of OA patients without synovial calcium crystals have synovial tissue fragments [23]. These findings together suggest a potential role of OA FLSs, synovium, and synovium fragments in the formation and deposition of calcium crystals. The involvement of FLSs or synovium in the formation and deposition of calcium crystals is also supported by the findings that calcium crystals were detected in the synovium of patients with other medical disorders that affected articular joints [24–26]. However, the influence of synovial cells or FLSs-mediated formation and deposition of calcium minerals have not been examined to date.

An early study examined arthritis patients who had no radiographic evidence of chondrocalcinosis; this work found that BCP crystals were present in 66% synovial fluid specimens derived from OA patients and in 20% synovial fluid specimens derived from RA patients [27]. Another early study found that alizarin staining was positive in 54% synovial fluid specimens derived from OA patients and in 39% synovial fluid specimens derived from RA patients [28]. A recent analysis found that the prevalence of synovial CPPD crystals was 22% in OA patients and 8% in RA patients [29]. Furthermore, studies found that the calcium content in the synovial fluid of OA patients was higher than the calcium content in the synovial fluid of RA patients and that calcium crystals were detected in 100% synovial fluid specimens derived from OA patients [3, 30].

These findings taken together suggest that OA synovial cells can play a role in the pathological calcification process associated with OA and may have a higher calcifying potential than RA synovial cells. In this study, we examined and compared the expression of genes implicated in the biomineral formation biological process in OA FLSs and RA FLSs, FLSs-mediated calcium mineral formation and deposition in the absence and presence of ATP in regular medium, and chondrogenesis differentiating medium and osteogenesis differentiation medium to test the hypothesis that OA FLSs are not bystanders but potentially active players in the pathological calcification process occurring in OA.

## 2. Materials and Methods

### 2.1. Materials

Dulbecco's minimum essential medium (DMEM), STEMPro chondrogenesis differentiation medium, STEMPro osteogenesis differentiation medium, fetal bovine serum (FBS), and stock antibiotic/antimycotic mixture were obtained from Invitrogen (Carlsbad, CA, USA). ^45^Calcium was obtained from Perkin-Elmer (Boston, MA, USA). Paraplast plus paraffin and eosin Y were obtained from Thermo Shandon Inc. (Pittsburgh, PA, USA). PC was prepared according to the methods previously described [31]. All other chemicals were obtained from Sigma (St. Louis, MO, USA).

### 2.2. Cell Cultures

hTERT-OA 13A FLSs and hTERT-RA 516 FLSs, telomerase-immortalized human OA FLSs, and RA FLSs have been described previously [21]. Primary OA FLSs were prepared from OA synovial tissues. Primary RA FLSs were a gift from Dr. Gary Firestein (University of California, San Diego, CA). OA FLSs and RA FLSs were maintained and expanded in DMEM containing 10% FBS and 0.5% antibiotic/antimycotic solution. OA synovial tissue specimens were obtained with the approval of the authors' Institutional Review Board from OA patients undergoing total joint replacement surgery at Carolinas Medical Center. Because these synovial tissue specimens were surgical waste from routine joint replacement surgeries and no private patient information was collected, the need for informed consent was waived.

### 2.3. RNA Extraction and Microarray

hTERT-OA 13A FLSs and hTERT-RA 516 FLSs were plated at 85–90% confluence in 100 mm plates. The next day, DMEM containing 10% FBS was added. Forty-eight hour later, total RNA was extracted using Trizol reagent (Qiagen, Valencia, CA) and subjected to microarray analysis. Briefly, double stranded DNA was synthesized using SuperScript double-stranded cDNA synthesis kit (Invitrogen, Carlsbad, CA). The DNA product was purified using GeneChip sample cleanup module (Affymetrix, Santa Clara, CA). cRNA was synthesized and biotin labeled using BioArray high yield RNA transcript labeling kit (Enzo Life Sciences, Farmingdale, NY). The cRNA product was purified using GeneChip sample cleanup module and subsequently chemically fragmented. The fragmented, biotinylated cRNA was hybridized to HG-U133_Plus_2 gene chip using Affymetrix Fluidics Station 400. Fluorescent signal was quantified during two scans using Agilent Gene Array Scanner G2500A (Agilent Technologies, Palo Alto, CA) and GeneChip operating Software (Affymetrix). Genesifter software (VizX Labs, Seattle, WA) was used for the analysis of fold changes.

### 2.4. Semiquantitative RT-PCR

RNA samples (1 *μ*g) were reverse transcribed at 60°C for 60 minutes, followed by enzyme inactivation at 85°C for 5 minutes. RT-PCR experiments were carried out using ThermoScript RT-PCR machine (Invitrogen). Amplifications were carried out for 30–40 cycles by denaturing at 95°C for 30 seconds, annealing at 55°C for 30 seconds, and extending at 72°C for 45 seconds, with a final extension at 72°C for 10 minutes. The expression of the housekeeping gene *β*-actin was used as an internal control. RT-PCR products were electrophoresed on 2% agarose gel stained with ethidium bromide and photographed using a low light image system (ChemiImager 4000, Alpha Innotech Corporation, San Leandro, CA). Each RT-PCR experiment was repeated three times.

### 2.5. Real Time RT-PCR

Briefly, cDNA was synthesized using TaqMan Reverse Transcription reagents (Applied Biosystems, Inc., University Park, IL). Quantification of relative transcript levels for selected genes was performed using ABI7000 Real Time PCR system (Applied Biosystems, Inc.). TaqMan Gene Expression assay (Applied Biosystems, Inc.) was used, with FAM-MGB probes for fluorescent detection. cDNA samples were amplified with an initial Taq DNA polymerase activation step at 95°C for 10 minutes, followed by 40 cycles of denaturation at 95°C for 15 seconds and annealing at 60°C for one minute. Fold changes were calculated and the expression levels of genes were normalized to the expression level of glyceraldehyde 3-phosphate dehydrogenase (GAPDH) according to the method described [32]. Each real time RT-PCR experiment was repeated twice (with specimens running in triplicate) and results were averaged.

### 2.6. ATP-Induced Calcium Mineral Formation Assay

Cell-mediated calcium mineral formation and deposition were first investigated using a well-characterized ATP-induced CPPD crystal formation assay. It has been demonstrated that ^45^Calcium uptake in the monolayer cultures of chondrocytes is proportional to CPPD crystal formation [33, 34]. Briefly, cells were plated in twenty-four-well plates at 95 to 100% confluence. The next day, culture media were replaced with serum-free DMEM. On the third day, the media were replaced with DMEM trace labeled with 1 *μ*Ci/mL ^45^Calcium. ATP was added immediately at a final concentration of 1 mM. Cells without ATP treatment or cells with* beta*-glycerophosphate treatment were used as controls. Forty-eight hours later, culture media were removed from the wells. Cells were washed with cold Hank's balanced salt solution five times and treated with 0.1 N NaOH. The radioactivity of the cell lysate in each well was quantified by liquid scintigraphy and normalized against total protein. Assays were run in triplicate and the results were expressed as mean ± SD.

### 2.7. Calcium Mineral Formation in the Absence of ATP

Because the ATP-induced calcium mineral formation assay was mainly used to detect ATP-induced formation of CPPD crystals, we decided to further examine the FLSs-mediated calcium mineral formation and deposition in the absence of ATP using alizarin red, a method which can detect all types of calcium crystals. Briefly, hTERT-OA 13A FLSs, hTERT-RA 516 FLSs, primary OA FLSs, and primary RA FLSs were plated in forty-eight well plates at 85–90% confluence. The next day, culture medium was replaced with STEMPro chondrogenesis differentiation medium or STEMPro osteogenesis differentiation medium (triplicate). The cells were cultured for 9 days and fed with fresh STEMPro chondrogenesis differentiation medium or STEMPro osteogenesis differentiation medium every three days. At the end of the experimental period, medium was removed. Calcium mineral formation and deposition were examined using alizarin red. Alizarin red stains were extracted from each well with 200 *μ*L acidic water (0.1 mM hydrogen chloride) and quantified by reading at 405 nm using a microplate reader. This experiment was repeated four times.

### 2.8. Calcium Mineral Deposition in Micromass Culture

Briefly, FLSs were harvested from 100 mm plates and suspended in DMEM containing 10% FBS. For preparation of a micromass, a droplet of the cell suspension containing 6 × 10^6^ cells was placed in a well of 24-well plate. After placement of all droplets, the plate was incubated for 4 hours in a 37°C culture incubator. These micromasses were then fed with STEMPro chondrogenesis differentiation medium without ATP or with ATP (triplicate) every three days throughout the experiment for 14 days.

At the end of the experimental period, each well containing a micromass was rinsed twice with 500 *μ*L of Hank's balanced salt solution. Two drops of eosin were added to each well. Five minutes later, eosin was aspirated off and the micromasses were transferred to a strip of filter paper on top of an ethanol-soaked sponge within a plastic histology specimen cassette. The cassettes sat in 10% formalin solution for one hour. These micromass specimens underwent routine paraffin embedding. Sections (three sections for each micromass) were cut at 5 *μ*m and stained with alizarin red. This micromass experiment was repeated three times.

### 2.9. Nonaqueous Alcoholic Eosin Staining

The nonaqueous alcoholic eosin staining method was used to detect birefringent crystals [35]. Briefly, the micromasses were fixed in 10% formalin solution for 8 hours and then processed for paraffin embedding. Sections (four sections for each micromass) were cut at 7 *μ*m thick and stained with 0.5% alcoholic eosin Y. These slides were examined under polarization optics on a light microscope.

### 2.10. Statistical Analysis

The results of calcium mineral formation assays were expressed as the mean ± SD. The difference between two groups was analyzed using Student's *t*-test. In all cases, *P* < 0.01 was considered significant. Statistical analysis was performed using the SAS software, version 9.3 (SAS Institute Inc., Cary, NC, USA).

## 3. Results

### 3.1. Expression of Genes Classified in Biomineral Formation

We recently reported that numerous genes implicated in the inflammatory response biological process are differentially expressed in hTERT-OA 13A FLSs and hTERT-RA 516 FLSs [21]. Here, we expanded the analysis and found that many genes classified in biomineral formation biological process were differentially expressed.

Of the 31 differentially expressed genes (fold changes >1.8), eight genes displayed elevated expressions and 23 genes displayed decreased expressions in the hTERT-OA 13A FLSs compared with the hTERT-RA 516 FLSs (Table 1). The genes displaying elevated expression included immunoglobulin superfamily member 10 (IGSF10: 10.97-fold), chordin-like 1 (CHRDL1: 8.40-fold), and twist homolog 2 (TWIST2: 5.21-fold). The genes displayed elevated expression also included runt-related transcription factor 2 (RunX2: 2.26-fold), ENPP1 (1.95-fold), and bone gamma-carboxyglutamate (gla) protein/osteocalcin (BGLAP/osteocalcin: 1.87-fold), all of which were previously implicated in OA or pathological calcification associated with OA [36–39].

The genes which displayed decreased expression in hTERT-OA 13A FLSs included fibroblast growth factor 9 (FGF9: −29.84-fold), prostaglandin E receptor 4 (PTGER4: −10.03-fold), ANKH (−5.26-fold), pleiotrophin (PTN: −4.7-fold), parathyroid hormone-like hormone (PTHLH: −3.6-fold), and serglycin (SRGN: −1.9-fold). The genes displaying decreased expression also included epidermal growth factor receptor (EGFR: −5.57-fold), connective tissue growth factor (CTGF: −4.99-fold), adrenergic beta-2 receptor (ADRB2: −4.45-fold), transforming growth factor beta 1 (TGF-*β*1: −2.16-fold), bone morphogenetic protein 6 (BMP6: −3.49-fold), and tumor necrosis factor receptor superfamily member 11a (TNFRSF11A/RANK: −2.35-fold). The majority of these genes have been previously implicated in RA [40–45].

**Figure 1:**
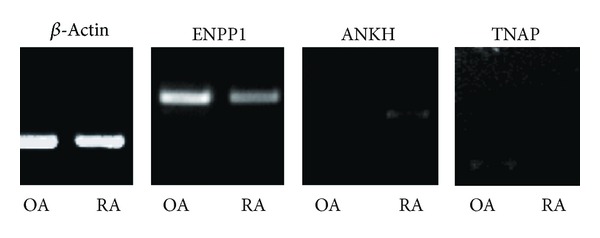
Semiquantitative RT-PCR. RNA samples were extracted from hTERT-OA 13A FLSs and hTERT-RA 516 FLSs. The messenger levels of ENPP1, ANKH, and TNAP were determined by RT-PCR. *β*-Actin, 30 cycles; ENPP1, 35 cycles; ANKH, 40 cycles; and TNAP, 35 cycles.

To confirm the differential expression of ENPP1 and ANKH, we performed semiquantitative RT-PCR. As shown in Figure 1, the elevated expression of ENPP1 and decreased expression of ANKH in hTERT-OA 13A FLSs were conformed. We also examined the expression of tissue-nonspecific alkaline phosphatase (TNAP) and found that the expression of TNAP was also elevated in hTERT-OA 13A FLSs (Figure 1).

We also performed real time RT-PCR experiments to confirm the differential expression of selected genes. The results are listed in Table 2. As shown, the elevated expression of RunX2, ENPP1, and BGLAP/osteocalcin and the decreased expression of PTN, CTGF, and SRGN were confirmed by real time RT-PCR.

### 3.2. FLSs-Mediated Calcium Formation and Deposition

**Figure 2:**
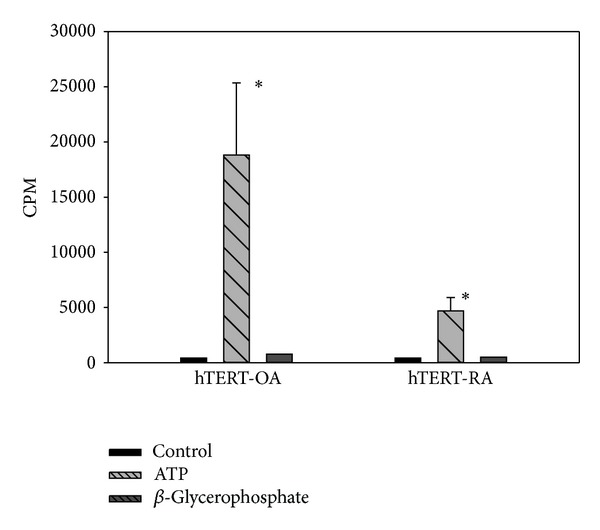
ATP-induced calcium mineral formation and deposition in monolayer culture. Left bar group: untreated hTERT-OA 13A FLSs, ATP-treated hTERT-OA 13A FLSs, and* beta*-glycerophosphate treated hTERT-OA 13A FLSs (from left to right). Right bar group: untreated hTERT-RA 516 FLSs, ATP-treated hTERT-RA 516 FLSs, and* beta*-glycerophosphate treated hTERT-RA 516 FLSs (from left to right). Counts per minute (CPM) were normalized against total protein levels.

First, we examined FLSs-mediated calcium mineral formation and deposition using an ATP-induced CPPD crystal formation and deposition assay. We found that ATP induced an ~40-fold increase in calcium mineral deposition in the monolayer culture of hTERT-OA 13A FLSs (Figure 2, the left bar group) and an ~10-fold increase in calcium mineral deposition in the monolayer culture of hTERT-RA 516 FLSs (Figure 2, the right bar group). This result indicated that FLSs were capable of mediating the formation of CPPD crystals and that the ATP-induced calcium mineral deposition mediated by hTERT-OA 13A FLSs (CPM=18,815) was ~4-fold greater than that mediated by hTERT-RA 516 FLSs (CPM=4,692). This difference is statistically significant (*P* < 0.005). When* beta*-glycerophosphate was used to replace ATP as an alternative source of phosphate, only a small amount of calcium minerals (less than two-fold increase) was formed and deposited (Figure 2).

**Figure 3:**
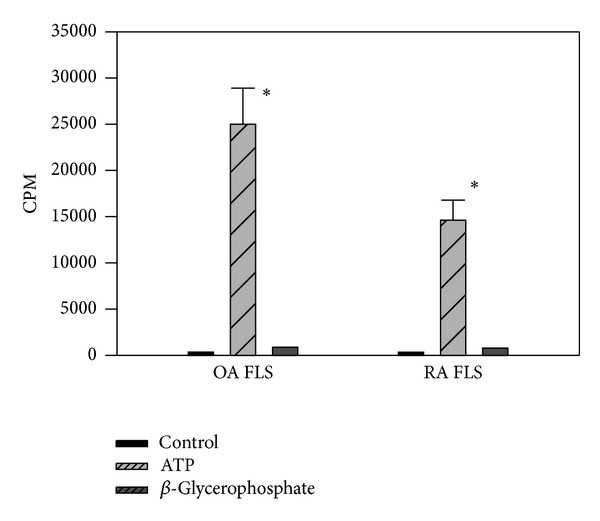
ATP-induced calcium mineral formation and deposition in monolayer culture of primary FLSs. Left panel: untreated OA FLSs, ATP-treated OA FLSs, and* beta*-glycerophosphate treated OA FLSs. Right panel: untreated RA FLSs, ATP-treated RA FLSs, and* beta*-glycerophosphate treated RA FLSs. Counts per minute (CPM) were normalized against total protein levels.

Next, we examined FLSs-mediated calcium mineral formation and deposition using primary OA FLSs derived from five OA patients (mean age 49 years) and primary RA FLSs derived from four RA patients (mean age 38 years). Consistently, we found that ATP-induced calcium mineral deposition (CPM=25,005) in the monolayer cultures of primary OA FLSs was collectively ~2-fold greater than that (CPM=14,606) in the monolayer cultures of primary RA FLSs (Figure 3). The difference is statistically significant (*P* < 0.005). Similarly, only a small amount of calcium mineral (less than twofold increase) was formed and deposited when* beta*-glycerophosphate was used to replace ATP. Detailed results are listed in Table 3.

### 3.3. Alizarin Red Staining

**Figure 4:**
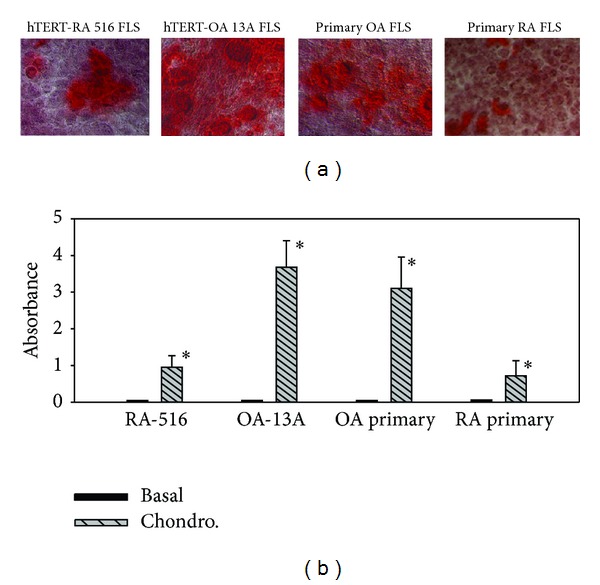
Alizarin red staining and reading. (a) Alizarin red staining of hTERT-RA 516 FLSs, hTERT-OA 13A FLSs, primary OA FLSs, and primary RA FLSs cultured in STEMPro chondrogenesis differentiation medium. (b) Absorbance of alizarin red extract from the monolayer cultures of hTERT-RA 516 FLSs, hTERT-OA 13A FLSs, primary OA FLSs, and primary RA FLSs cultured in the basal medium and STEMPro chondrogenesis differentiation medium.

To further investigate OA FLSs- and RA FLS-mediated calcium mineral formation and deposition, we cultured OA FLSs and RA FLSs in STEMPro chondrogenesis differentiation medium for 14 days in the absence of ATP and examined calcium mineral formation and deposition using alizarin red. Representative alizarin red staining and readings are shown in Figure 4. Consistently, we found more alizarin stains in the monolayer culture of hTERT-OA 13A FLSs than in the monolayer culture of hTERT-RA 516 FLSs (Figure 4(a)). The experiment was also performed using primary OA FLSs and RA FLSs, and similar results were obtained (Figure 4(a)).

The alizarin red was extracted and quantified by reading at 405 nm. As shown in Figure 4(b), ~4–6 times more alizarin red was detected in the extracts from the monolayer cultures of hTERT-OA 13A FLSs and primary OA FLSs than in the extracts from the monolayer cultures of hTERT-RA 516 FLSs and primary RA FLSs (*P* < 0.01).

**Figure 5:**
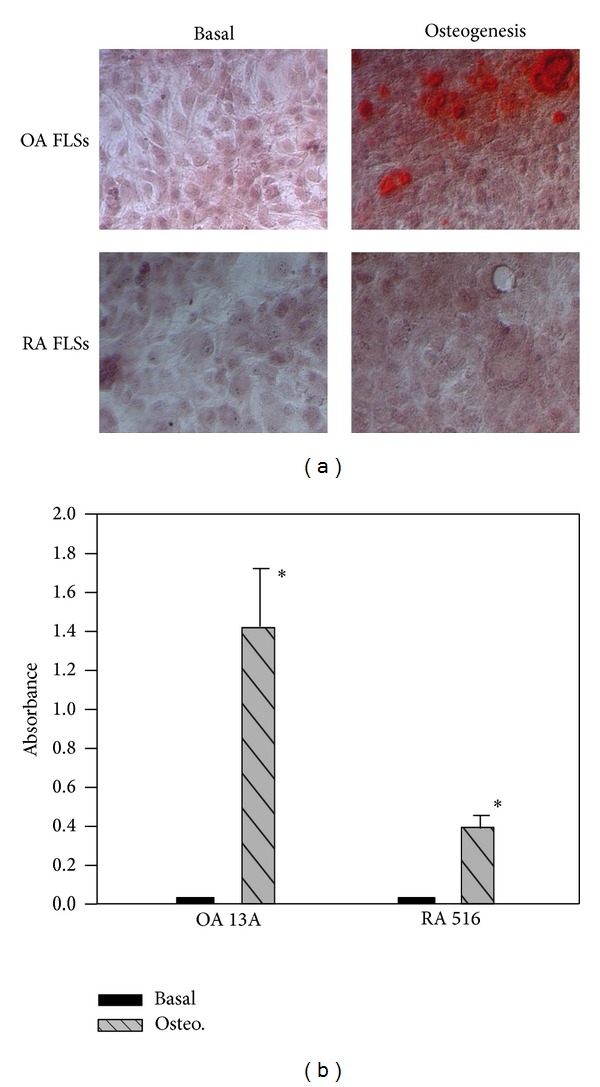
Alizarin red staining and analysis.(a) Alizarin red staining of hTERT-OA 13A FLSs and hTERT-RA 516 FLSs cultured in basal medium and STEMPro osteogenesis differentiation medium. (b)Absorbance of alizarin red extract from the monolayer cultures of hTERT-OA 13A FLSs and hTERT-RA 516 FLSs cultured in basal medium and STEMPro osteogenesis differentiation medium.

We also cultured hTERT-OA 13A FLSs and hTERT-RA 516 FLSs in STEMPro osteogenesis differentiation medium in the absence of ATP and examined calcium mineral formation and deposition using alizarin red. Representative alizarin red staining and results are shown in Figure 5. Again, we found more alizarin stains in the monolayer culture of hTERT-OA 13A FLSs than in the monolayer culture of hTERT-RA 516 FLSs. However, it was clear that hTERT-OA 13A FLSs and hTERT-RA 516 FLSs cultured in the STEMPro osteogenesis differentiation medium produced less calcium mineral than the hTERT-OA 13A FLSs and hTERT-RA 516 FLSs cultured in the STEMPro chondrogenesis differentiation medium (comparing Figure 5 with Figure 4). This finding suggests that the differentiation of FLSs into chondrocyte-like cells, but not osteoblast-like cells, may play a major role in the FLSs-mediated calcium mineral formation and deposition.

### 3.4. Calcium Mineral Deposition in Micromass Culture

**Figure 6:**
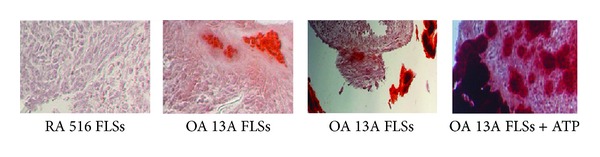
Alizarin red staining. Micromass of hTERT-RA 516 FLSs cultured in the absence of ATP. There were no calcium deposits detected (first photo from the left). Micromass of hTERT-OA 13A FLSs cultured in the absence of ATP. Small amounts of calcium deposits were detected within the micromass (second photo from the left). Micromass of hTERT-OA 13A FLSs cultured in the absence of ATP. Large amounts of calcium deposits were detected in the fragments of the micromass, but not within the micromass (third photo from the left). Micromass of hTERT-OA 13A FLSs cultured in the presence of ATP. Large amounts of calcium deposits were detected within the micromass (far right photo).

We cultured the micromasses of hTERT-OA 13A FLSs and hTERT-RA 516 FLSs in STEMPro chondrogenesis differentiation medium for 14 days and assessed calcium mineral formation and deposition using alizarin red staining. Representative images of alizarin red staining are shown in Figure 6. As shown, no calcium mineral deposits were detected in the micromass of hTERT-RA FLSs cultured in the absence of ATP (left photo) whereas small amounts of calcium mineral deposits were detected in the micromass of hTERT-OA 13A FLSs (second photo from the left). Calcium mineral deposits were also detected in the fragments of the micromass of hTERT-OA 13A FLSs (third photo from the left), indicating that synovial fragments may play a role in pathological calcification. Certain unknown factors (such as collagen breaking ends) may promote the formation of calcium minerals within the synovial fragments. In the presence of ATP, large amounts of calcium mineral deposits were detected in the micromass of hTERT-OA 13A FLSs. Similar results were obtained in the micromasses of primary OA FLSs (data not shown).

### 3.5. Detection of Birefringent Crystals

**Figure 7:**
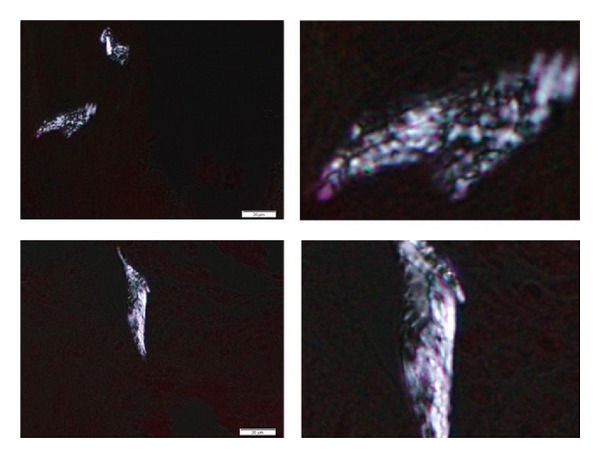
Nonaqueous alcoholic eosin staining. Birefringent crystals in the micromasses of hTERT-OA 13A FLSs were observed using polarizing light microscope (left photos). Enlarged images of the respective birefringent crystals are shown in the corresponding right hand images.

We examined the micromasses of hTERT-OA 13A FLSs using nonaqueous alcoholic eosin staining method. Representative images of the stained sections are shown in Figure 7. Under polarizing light microscopy, the presence of birefringent crystals was confirmed. These birefringent crystals were aggregates of rhomboid- and rod-shaped crystals. The micromass experiment was repeated three times (3 micromasses/experiment) and the presence of birefringent crystals was detected in 3 micromasses among the 9 micromasses examined.

### 3.6. PC Inhibits FLSs-Mediated Calcium Mineral Formation and Deposition

**Figure 8:**
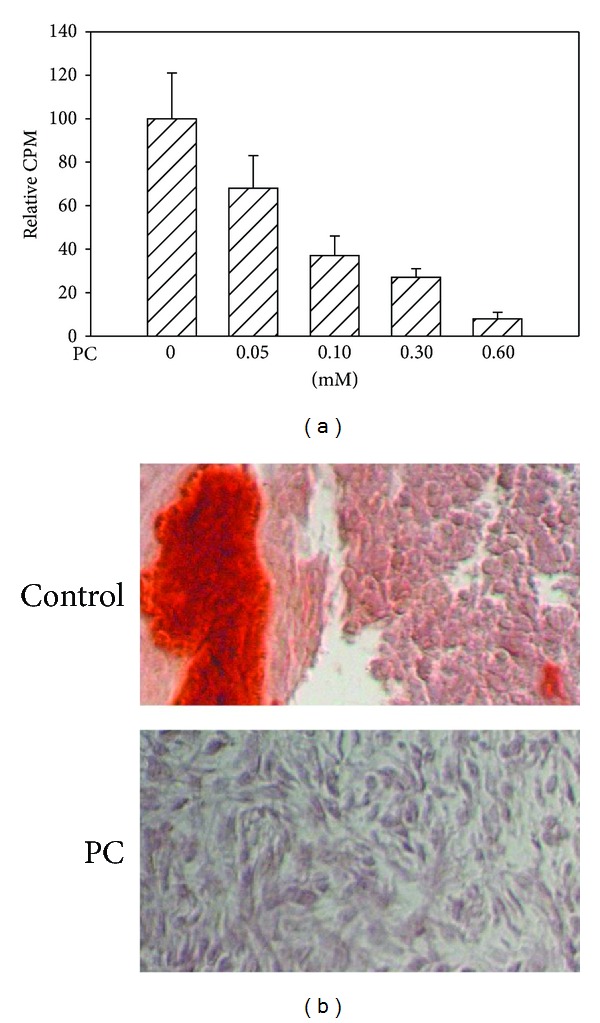
Inhibition of calcium mineral formation and deposition. (a) PC inhibited ATP-induced calcium mineral formation and deposition in the monolayer culture of OA FLSs in a dose dependent manner (*P* < 0.001) and calcium mineral formation and deposition were completely abolished by 0.6 mM PC. (b) Calcium mineral formation and deposition in the micromass culture of OA FLSs were completely abolished by 0.6 mM PC.

When performing ATP-induced calcium deposition assay, increased amounts of PC were added in select wells. Twenty-four hours later, calcium deposition was examined. The results are shown in Figure 8(a). PC inhibited OA FLSs-mediated calcium mineral formation and deposition in a dose dependent manner and calcium mineral formation and deposition were completely abolished by 0.6 mM PC. Similarly, calcium mineral formation and deposition in the micromass culture of OA FLSs were completely abolished by 0.6 mM PC (Figure 8(b)).

## 4. Discussion

The presence of BCP crystals in the synovial fluid of OA patients is well recognized; however, the sources of these crystals remain unclear. In this study, we found that many genes classified in the biomineral formation process were differently expressed in the hTERT-OA 13A FLSs and hTERT-RA 516 FLSs (Table 1). The findings that the expression level of BGLAP/osteocalcin gene is higher and the expression levels of BMP6, TGF-*β*1, PTHLH, and PTN genes are lower in the hTERT-OA 13A FLSs compared to hTERT-RA 516 FLSs are consistent with the findings that the protein level of BGLAP/osteocalcin is significantly higher [46] and the protein levels of BMP6, PTHLH, PTN, and TGF-*β*1 are significantly lower in the synovial fluid of OA patients compared to RA patients [43, 45, 47–49].

Many of the genes displaying lower expression levels in the hTERT-OA 13A FLSs have been previously implicated in the negative regulation of biomineral formation. For example, SRGN inhibited the growth of hydroxyapatite crystals [50]; PTHLH and FGF9 inhibited terminal differentiation of chondrocytes and mineralization [51–53]; mutation of ANKH caused chondrocalcinosis [54, 55]; CTGF-treated mesenchymal stem cells (MSCs) lost the ability to differentiate into chondrocytes and osteoblasts [56]; EGFR signaling suppressed osteoblast differentiation [57, 58]; and PTGER4 mediated the inhibition of mineralization in mature cementoblasts by prostaglandin E2 [59]. In contrast, many of the genes displaying higher expression levels have been previously implicated in the positive regulation of biomineral formation. For example, BGLAP/osteocalcin induced cartilage calcification [39], forced overexpression of Runx2 or TNAP, upregulated osteoblast-specific genes, and enhanced mineralization [60, 61]. These findings together suggest that OA FLSs may have a higher calcifying potential than RA FLSs and they may respond to calcifying stimuli differently.

Indeed, we found that FLSs were capable of mediating calcium mineral formation and deposition and that OA FLSs had a higher calcifying potential than RA FLSs. In addition, we found that the majority of calcium minerals formed in FLSs micromass culture were nonbirefringent. These findings are consistent with the clinical observations that calcium crystals are present in the synovial fluid and synovial membranes of OA patients and the majority of crystals presented in the OA synovial fluid are BCP crystals [1, 2]. These findings are also consistent with the clinical observations that calcium crystals are present within the knee joints of all OA patients but only in 20–39% RA patients [27–30]. Importantly, these findings are significant and indicate that OA FLSs are not bystanders but are rather potentially active players in the pathological calcification process occurring in OA. These crystals can interact with the synovium and induce mitogenesis and angiogenesis leading to synovial hyperplasia or interact with synovium, menisci, and cartilage to stimulate the production of MMPs and inflammatory cytokines leading to further degeneration of articular cartilage and menisci.

Previous studies demonstrated that CHRDL1 enhanced the proliferation of MSCs [62]. IGSF10 was a unique marker of early osteochondral progenitor cells [63] and TWIST2 played a role to maintain MSCs in preosteoblast-like state [64, 65]. The higher expression levels of CHRD1, IGSF10, and TWIST2 in the hTERT-OA 13A FLSs compared to hTERT-RA 516 FLSs suggest that hTERT-OA 13A FLSs may possess certain MSCs-like properties. Under pathological conditions, a small number of these OA FLSs may differentiate into chondrocyte-like or osteoblast-like cells and produce calcium minerals. To test this, we decided to examine the FLSs-mediated calcium mineral formation and deposition under conditions that promote chondrogenesis differentiation or osteogenesis differentiation. Consistently, we found that more calcium mineral was formed and deposited in OA FLSs cultured in the chondrogenesis differentiation medium or osteogenesis differentiation medium than in OA FLSs cultured in the basal medium. These findings indicate that OA FLSs differentiation may play a role in calcium mineral formation and deposition, which is consistent with the previous findings that intra-articular injected synovial cells and implanted autologous graft of synovial tissue differentiate into fibrocartilage cells and produce calcium crystals [66, 67].

We found that more calcium minerals were formed in the monolayer cultures of OA FLSs and RA FLSs cultured in the STEMPro chondrogenesis differentiation medium than in the monolayer cultures of OA and RA FLSs cultured in STEMPro osteogenesis differentiation medium. This finding indicates that the differentiation of OA FLSs into hypertrophic chondrocytes-like cells, but not osteoblast-like cells, may play an important role in the OA FLSs-mediated calcium mineral formation and deposition.

The finding that ATP stimulated more calcium mineral formation and deposition in OA FLSs than in RA FLSs, together with the previous finding that OA synovial fluid contains higher concentrations of nucleoside triphosphate pyrophosphatase and ATP than RA synovial fluid [68, 69], provides an explanation for the prevalence of calcium crystals in the synovial fluid of OA patients but not in the synovial fluid of RA patients. These findings also indicate that ATP is likely a major calcification stimulus for the OA FLSs-mediated pathological calcification process.

The finding that more calcium minerals are formed and deposited in chondrogenesis condition, together with the previous finding that the protein level of BGLAP/osteocalcin is significantly higher in the OA synovial fluid than that in RA synovial fluid [46], suggests a potential role of FLSs differentiation-inducing factors in the OA FLSs-medicated pathological calcification process. Our finding that the expressions of BGLAP/osteocalcin and Runx2 genes were higher in the hTERT-OA 13A FLSs compared to hTERT-RA FLSs is consistent with this new mechanism. Previous studies demonstrated that BGLAP and Runx2 promoted calcification [39, 60].

Finally, we demonstrate that PC significantly inhibits the OA FLSs-mediated calcium mineral formation and deposition. Interestingly, the morphology of OA FLSs (round and chondrocyte-like) within the micromasses in the absence of PC is different from the morphology of OA FLSs (long and fibroblast-like) in the presence of PC (Figure 8(b)). This phenomenon indicates that PC may prevent OA FLSs differentiation through an unknown mechanism and, in doing so, inhibits FLSs-mediated pathological calcification process in addition to the well-known mechanism or its direct binding to amorphous calcium-phosphate aggregates and crystals.

## 5. Conclusions

Our study suggests that OA FLSs are not passive bystanders, but active players in the pathological calcification process occurring in OA. ATP and certain other unidentified differentiation-inducing factors presented within OA synovial fluid may play an important role in the OA FLSs-mediated calcium mineral formation and deposition. The FLSs-mediated calcification process is a valid target for the development of disease-modifying drug for OA therapy.

## Figures and Tables

**Table 1:** Genes differentially expressed in hTERT-OA 13A FLSs compared with hTERT-RA 516 FLSs.

Biological process	Gene name	Differential expression (fold)*	Description
Biomineral formation	IGSF10	10.97	Immunoglobulin superfamily, member 10
	CHRDL1	8.40	Chordin-like 1
	TWIST2	5.21	Twist homolog 2 (Drosophila)
	RUNX2	2.26	Runt-related transcription factor 2
	ENPP1	1.95	Ectonucleotide pyrophosphatase/phosphodiesterase 1
	BGLAP	1.87	Bone gamma-carboxyglutamate (gla) protein (osteocalcin)
	BMPR1B	1.87	Bone morphogenetic protein receptor, type IB
	COL13A1	1.85	Collagen, type XIII, alpha 1
	FGF9	−29.84	Fibroblast growth factor 9 (glia-activating factor)
	PTGER4	−10.03	Prostaglandin E receptor 4 (subtype EP4)
	EGFR	−5.57	Epidermal growth factor receptor
	ANKH	−5.26	Ankylosis, progressive homolog (mouse)
	CTGF	−4.99	Connective tissue growth factor
	PTN	−4.67	Pleiotrophin
	ADRB2	−4.45	Adrenergic, beta-2-, receptor, surface
	TGFB2	−4.12	Transforming growth factor, beta 2
	TGFB3	−3.07	Transforming growth factor, beta 3
	TGFB1	−2.16	Transforming growth factor, beta 1
	PTHLH	−3.61	Parathyroid hormone-like hormone
	BMP6	−3.49	Bone morphogenetic protein 6
	BMP4	−2.86	Bone morphogenetic protein 4
	MEF2C	−3.18	Myocyte enhancer factor 2C
	CDK6	−3.13	Cyclin-dependent kinase 6
	FOXC1	−3.02	Forkhead box C1
	TNFRSF11A	−2.35	Tumor necrosis factor receptor superfamily, member 11a
	AXIN2	−2.31	Axin 2 (conductin, axil)
	GNAS	−2.13	GNAS complex locus
	SORT1	−2.00	Sortilin 1
	TUFT1	−1.91	Tuftelin 1
	SRGN	−1.90	Serglycin
	GLI2	−1.82	GLI-Kruppel family member GLI2

*Positive number indicates elevated expression (fold) in hTERT-OA 13A FLSs compared with hTERT-RA 516 FLSs.

Negative number indicates decreased expression (fold) in hTERT-OA 13A FLSs compared with hTERT-RA 516 FLSs.

**Table 2:** Differential expression detected by microarray and real time RT-PCR*.

Gene name	Microarray	Real time RT-PCR	Description
RunX2	2.26	2.5	Runt-related transcription factor 2
ENPP1	1.95	2.4	Ectonucleotide pyrophosphatase/phosphodiesterase 1
BGLAP	1.87	2.1	Bone gamma-carboxyglutamate (gla) protein (osteocalcin)
PTN	−4.67	−5.2	Pleiotrophin
CTCG	−4.99	−2.8	Connective tissue growth factor
SRGN	−1.90	−1.7	Serglycin

*Positive number indicates elevated expression (fold) in hTERT-OA 13A FLSs compared with hTERT-RA 516 FLSs.

Negative number indicates decreased expression (fold) in hTERT-OA 13A FLSs compared with hTERT-RA 516 FLSs.

**Table 3:** Primary OA FLSs-mediated and primary RA FLSs-mediated calcium mineral deposition.

OA FLSs			RA FLSs		
Age/gender of patients	Age/gender of patients	Age/gender of patients	Age/gender of patients	Control (CPM)	ATP (CPM)
42 M	27 F	27 F	27 F	375 ± 91	28,580 ± 2,825
48 F	39 F	39 F	39 F	345 ± 123	19,790 ± 1005
50 F	42 F	42 F	42 F	290 ± 48	24,155 ± 1,987
52 F	43 M	43 M	43 M	365 ± 56	24,310 ± 1,105
54 F				490 ± 89	28,325 ± 1,850

F: female; M: male; CMP: count per minute normalized against total protein levels; control—cultured in the absence of ATP; ATP—cultured in the presence of ATP.

## References

[1] Derfus BA, Kurian JB, Butler JJ (2002). The high prevalence of pathologic calcium crystals in pre-operative knees. *Journal of Rheumatology*.

[2] Nalbant S, Martinez JAM, Kitumnuaypong T, Clayburne G, Sieck M, Schumacher HR (2003). Synovial fluid features and their relations to osteoarthritis severity: new findings from sequential studies. *Osteoarthritis and Cartilage*.

[3] Nero P, Nogueira I, Vilar R, Pimentão JB, Branco JC (2006). Synovial fluid crystal identification by electron microscopy. *Acta Reumatologica Portuguesa*.

[4] Fuerst M, Bertrand J, Lammers L (2009). Calcification of articular cartilage in human osteoarthritis. *Arthritis and Rheumatism*.

[5] Fuerst M, Niggemeyer O, Lammers L, Schäfer F, Lohmann C, Rüther W (2009). Articular cartilage mineralization in osteoarthritis of the hip. *BMC Musculoskeletal Disorders*.

[6] Sun Y, Mauerhan DR, Honeycutt PR (2010). Calcium deposition in osteoarthritic meniscus and meniscal cell culture. *Arthritis Research and Therapy*.

[7] McCarty DJ (1970). Crystal-induced inflammation of the joints. *Annual Review of Medicine*.

[8] Ea HK, Chobaz V, Nguyen C (2013). Pathogenic role of basic calcium phosphate crystals in destructive arthropathies. *PLoS ONE*.

[9] Morgan MP, Whelan LC, Sallis JD, McCarthy CJ, Fitzgerald DJ, McCarthy GM (2004). Basic calcium phosphate crystal-induced prostaglandin E2 production in human fibroblasts: role of cyclooxygenase 1, cyclooxygenase 2, and interleukin-1*β*. *Arthritis and Rheumatism*.

[10] McCarthy GM, Cheung HS, Abel SM, Ryan LM (1998). Basic calcium phosphate crystal-induced collagenase production: role of intracellular crystal dissolution. *Osteoarthritis and Cartilage*.

[11] Sun Y, Wenger L, Brinckerhoff CE, Misra RR, Cheung HS (2002). Basic calcium phosphate crystals induce matrix metalloproteinase-1 through the Ras/mitogen-activated protein kinase/c-Fos/AP-1/metalloproteinase 1 pathway: involvement of transcription factor binding sites AP-1 and PEA-3. *Journal of Biological Chemistry*.

[12] Ea H-K, Uzan B, Rey C, Lioté F (2005). Octacalcium phosphate crystals directly stimulate expression of inducible nitric oxide synthase through p38 and JNK mitogen-activated protein kinases in articular chondrocytes. *Arthritis Research & Therapy*.

[13] McCarthy GM, Mitchell PG, Cheung HS (1991). The mitogenic response to stimulation with basic calcium phosphate crystals is accompanied by induction and secretion of collagenase in human fibroblasts. *Arthritis and Rheumatism*.

[14] Cheung HS, Sallis JD, Demadis KD, Wierzbicki A (2006). Phosphocitrate blocks calcification-induced articular joint degeneration in a guinea pig model. *Arthritis and Rheumatism*.

[15] Cheung HS (2001). Phosphocitrate as a potential therapeutic strategy for crystal deposition disease. *Current Rheumatology Reports*.

[16] Saito I, Koshino T, Nakashima K, Uesugi M, Saito T (2002). Increased cellular infiltrate in inflammatory synovia of osteoarthritic knees. *Osteoarthritis and Cartilage*.

[17] Smith MD, Triantafillou S, Parker A, Youssef PP, Coleman M (1997). Synovial membrane inflammation and cytokine production in patients with early osteoarthritis. *Journal of Rheumatology*.

[18] Pearle AD, Scanzello CR, George S (2007). Elevated high-sensitivity C-reactive protein levels are associated with local inflammatory findings in patients with osteoarthritis. *Osteoarthritis and Cartilage*.

[19] Kato H, Matsumine A, Wakabayashi T (2007). Large-scale gene expression profiles, differentially represented in osteoarthritic synovium of the knee joint using cDNA microarray technology. *Biomarkers*.

[20] Galligan CL, Baig E, Bykerk V, Keystone EC, Fish EN (2007). Distinctive gene expression signatures in rheumatoid arthritis synovial tissue fibroblast cells: correlates with disease activity. *Genes and Immunity*.

[21] Sun Y, Mauerhan DR, Firestein GS, Loeffler BJ, Hanley EN, Gruber HE (2009). Telomerase transduced osteoarthritis fibroblast-like synoviocytes display a distinct gene expression profile. *Journal of Rheumatology*.

[22] Lagier R, Baud C-A, Lacotte D, Gerster J-C (1989). Osteoarthrosis and apatite synovitis: pathological study of a metacarpophalangeal joint. *Virchows Archiv A: Pathological Anatomy and Histopathology*.

[23] Dai L, Pessler F, Chen LX, Clayburne G, Schumacher HR (2006). Detection and initial characterization of synovial lining fragments in synovial fluid. *Rheumatology*.

[24] Halverson PB, Garancis JC, McCarthy DJ (1984). Histopathological and ultrastructural studies of synovium in Milwaukee shoulder syndrome—a basic calcium phosphate crystal arthropaty. *Annals of the Rheumatic Diseases*.

[25] Brandt KD, Krey PR (1977). Chalky joint effusion: the result of massive synovial deposition of calcium apatite in progressive systemic sclerosis. *Arthritis and Rheumatism*.

[26] Reginato AJ, Schumacher HR (1977). Synovial calcification in a patient with collagen-vascular disease: light and electron microscopic studies. *Journal of Rheumatology*.

[27] Swan A, Chapman B, Heap P, Seward H, Dieppe P (1994). Submicroscopic crystals in osteoarthritic synovial fluids. *Annals of the Rheumatic Diseases*.

[28] Hamilton E, Pattrick M, Hornby J, Derrick G, Doherty M (1990). Synovial fluid calcium pyrophosphate dihydrate crystals and alizarin red positivity: analysis of 3000 samples. *British Journal of Rheumatology*.

[29] Oliviero F, Scanu A, Galozzi P (2013). Prevalence of calcium pyrophosphate and monosodium urate crystals in synovial fluid of patients with previously diagnosed joint diseases. *Joint Bone Spine*.

[30] Yavorskyy A, Hernandez-Santana A, Shortt B, McCarthy G, McMahon G (2010). Determination of calcium in synovial fluid samples as an aid to diagnosing osteoarthritis. *Bioanalysis*.

[31] Turhanen PA, Demadis KD, Peräniemi S, Vepsäläinen JJ (2007). A novel strategy for the preparation of naturally occurring phosphocitrate and its partially esterified derivatives. *Journal of Organic Chemistry*.

[32] Pfaffl M, Meyer HHD, Sauerwein H (1998). Quantification of insulin-like growth factor-1 (IGF-1) mRNA: development and validation of an internally standardised competitive reverse transcription-polymerase chain reaction. *Experimental and Clinical Endocrinology and Diabetes*.

[33] Rosenthal AK, Gohr CM, Uzuki M, Masuda I (2007). Osteopontin promotes pathologic mineralization in articular cartilage. *Matrix Biology*.

[34] Rosenthal AK, Mattson E, Gohr CM, Hirschmugl CJ (2008). Characterization of articular calcium-containing crystals by synchrotron FTIR. *Osteoarthritis and Cartilage*.

[35] Shidham V, Chivukula M, Basir Z, Shidham G (2001). Evaluation of crystals in formalin-fixed, paraffin-embedded tissue sections for the differential diagnosis of pseudogout, gout, and tumoral calcinosis. *Modern Pathology*.

[36] Wang X, Manner PA, Horner A, Shum L, Tuan RS, Nuckolls GH (2004). Regulation of MMP-13 expression by RUNX2 and FGF2 in osteoarthritic cartilage. *Osteoarthritis and Cartilage*.

[37] Kamekura S, Kawasaki Y, Hoshi K (2006). Contribution of runt-related transcription factor 2 to the pathogenesis of osteoarthritis in mice after induction of knee joint instability. *Arthritis and Rheumatism*.

[38] Johnson K, Pritzker K, Goding J, Terkeltaub R (2001). The nucleoside triphosphate pyrophosphohydrolase isozyme PC-1 directly promotes cartilage calcification through chondrocyte apoptosis and increased calcium precipitation by mineralizing vesicles. *Journal of Rheumatology*.

[39] Idelevich A, Rais Y, Monsonego-Ornan E (2011). Bone Gla protein increases HIF-1*α*-dependent glucose metabolism and induces cartilage and vascular calcification. *Arteriosclerosis, Thrombosis, and Vascular Biology*.

[40] Lo SF, Wan L, Lin HC (2012). Association of rheumatoid arthritis risk with EGFR genetic polymorphisms in Taiwan’s Han Chinese population. *Rheumatology International*.

[41] Wang JG, Xu WD, Zhai WT (2012). Disorders in angiogenesis and redox pathways are main factors contributing to the progression of rheumatoid arthritis: a comparative proteomic study. *Arthritis and Rheumatology*.

[42] Baerwald C, Graefe C, Muhl C, von Wichert P, Krause A (1992). *β*2-Adrenergic receptors on peripheral blood mononuclear cells in patients with rheumatic diseases. *European Journal of Clinical Investigation*.

[43] Lories RJU, Derese I, Ceuppens JL, Luyten FP (2003). Bone morphogenetic proteins 2 and 6, expressed in arthritic synovium, are regulated by proinflammatory cytokines and differentially modulate fibroblast-Like synoviocyte apoptosis. *Arthritis and Rheumatism*.

[44] Poubelle PE, Chakravarti A, Fernandes MJ, Doiron K, Marceau A-A (2007). Differential expression of RANK, RANK-L, and osteoprotegerin by synovial fluid neutrophils from patients with rheumatoid arthritis and by healthy human blood neutrophils. *Arthritis Research and Therapy*.

[45] Schlaak JF, Pfers I,  Meyer Zum Büschenfelde KH, Märker-Hermann E (1996). Different cytokine profiles in the synovial fluid of patients with osteoarthritis, rheumatoid arthritis and seronegative spondylarthropathies. *Clinical and Experimental Rheumatology*.

[46] Mattei JP, Ferrera V, Boutsen Y (1992). Serum and synovial fluid osteocalcin in rheumatic diseases. *International Journal of Clinical Pharmacology Research*.

[47] Horiuchi T, Yoshida T, Koshihara Y (1999). The increase of parathyroid hormone-related peptide and cytokine levels in synovial fluid of elderly rheumatoid arthritis and osteoarthritis. *Endocrine Journal*.

[48] Kohno H, Shigeno C, Kasai R (1997). Synovial fluids from patients with osteoarthritis and rheumatoid arthritis contain high levels of parathyroid hormone-related peptide. *Journal of Bone and Mineral Research*.

[49] Pufe T, Groth G, Goldring MB, Tillmann B, Mentlein R (2007). Effects of pleiotrophin, a heparin-binding growth factor, on human primary and immortalized chondrocytes. *Osteoarthritis and Cartilage*.

[50] Theocharis AD, Seidel C, Borset M (2006). Serglycin constitutively secreted by myeloma plasma cells is a potent inhibitor of bone mineralization *in vitro*. *Journal of Biological Chemistry*.

[51] Jiang J, Leong NL, Mung JC, Hidaka C, Lu HH (2008). Interaction between zonal populations of articular chondrocytes suppresses chondrocyte mineralization and this process is mediated by PTHrP. *Osteoarthritis and Cartilage*.

[52] Schipani E, Lanske B, Hunzelman J (1997). Targeted expression of constitutively active receptors for parathyroid hormone and parathyroid hormone-related peptide delays endochondral bone formation and rescues mice that lack parathyroid hormone-related peptide. *Proceedings of the National Academy of Sciences of the United States of America*.

[53] Weksler NB, Lunstrum GP, Reid ES, Horton WA (1999). Differential effects of fibroblast growth factor (FGF) 9 and FGF2 on proliferation, differentiation and terminal differentiation of chondrocytic cells *in vitro*. *Biochemical Journal*.

[54] Williams CJ, Pendleton A, Bonavita G (2003). Mutations in the amino terminus of ANKH in two US families with calcium pyrophosphate dihydrate crystal deposition disease. *Arthritis and Rheumatism*.

[55] Netter P, Bardin T, Bianchi A, Richette P, Loeuille D (2004). The ANKH gene and familial calcium pyrophosphate dihydrate deposition disease. *Joint Bone Spine*.

[56] Lee CH, Shah B, Moioli EK, Mao JJ (2010). CTGF directs fibroblast differentiation from human mesenchymal stem/stromal cells and defines connective tissue healing in a rodent injury model. *Journal of Clinical Investigation*.

[57] Laflamme C, Curt S, Rouabhia M (2010). Epidermal growth factor and bone morphogenetic proteins upregulate osteoblast proliferation and osteoblastic markers and inhibit bone nodule formation. *Archives of Oral Biology*.

[58] Zhu J, Shimizu E, Zhang X, Partridge NC, Qin L (2011). EGFR signaling suppresses osteoblast differentiation and inhibits expression of master osteoblastic transcription factors Runx2 and Osterix. *Journal of Cellular Biochemistry*.

[59] Oka H, Miyauchi M, Sakamoto K (2008). Prostaglandin E2 inhibits mineralization and enhances matrix metalloproteinase-13 in mature cementoblasts mainly via the EP4 pathway. *Archives of Oral Biology*.

[60] Byers BA, García AJ (2004). Exogenous Runx2 expression enhances in vitro osteoblastic differentiation and mineralization in primary bone marrow stromal cells. *Tissue Engineering*.

[61] Narisawa S, Yadav MC, Millan JL (2013). *In vivo* overexpression of tissue-nonspecific alkaline phosphatase increases skeletal mineralization and affects the phosphorylation status of osteopontin. *Journal of Bone and Mineral Research*.

[62] Fernandes H, Dechering K, van Someren E (2010). Effect of chordin-like 1 on MC3T3-E1 and human mesenchymal stem cells. *Cells Tissues Organs*.

[63] Segev O, Samach A, Faerman A (2004). CMF608 -a novel mechanical strain-induced bone-specific protein expressed in early osteochondroprogenitor cells. *Bone*.

[64] Tamura M, Noda M (1999). Identification of DERMO-1 as a member of helix-loop-helix type transcription factors expressed in osteoblastic cells. *Journal of Cellular Biochemistry*.

[65] Lee MS, Lowe G, Flanagan S, Kuchler K, Glackin CA (2000). Human dermo-1 has attributes similar to twist in early bone development. *Bone*.

[66] Horie M, Sekiya I, Muneta T (2009). Intra-articular injected synovial stem cells differentiate into meniscal cells directly and promote meniscal regeneration without mobilization to distant organs in rat massive meniscal defect. *Stem Cells*.

[67] Stein H, Bab IA, Sela J (1981). The occurrence of hydroxyapatite crystals in extracellular matrix vesicles after surgical manipulation of the rabbit knee joint. *Cell and Tissue Research*.

[68] Pattrick M, Hamilton E, Hornby J, Doherty M (1991). Synovial fluid pyrophosphate and nucleoside triphosphate pyrophosphatase: comparison between normal and diseased and between inflamed and non-inflamed joints. *Annals of the Rheumatic Diseases*.

[69] Ryan LM, Rachow JW, McCarty DJ (1991). Synovial fluid ATP: a potential substrate for the production of inorganic pyrophosphate. *Journal of Rheumatology*.

